# Optimizing data visualization for reproductive, maternal, newborn, child health, and nutrition (RMNCH&N) policymaking: data visualization preferences and interpretation capacity among decision-makers in Tanzania

**DOI:** 10.1186/s41256-019-0095-1

**Published:** 2019-02-15

**Authors:** Tricia Aung, Debora Niyeha, Shagihilu Shagihilu, Rose Mpembeni, Joyceline Kaganda, Ashley Sheffel, Rebecca Heidkamp

**Affiliations:** 1 Department of International Health, Johns Hopkins Bloomberg School of Public Health, Baltimore, MD USA; 2 National Bureau of Statistics, Dar es Salaam, Tanzania; 3 School of Public Health and Social Sciences, Muhimbili University of Health and Allied Sciences, Dar es Salaam, Tanzania; 4 Tanzania Food and Nutrition Centre, Dar es Salaam, Tanzania

**Keywords:** Reproductive, Maternal, Newborn, Child health, Nutrition, Data visualization, Policy, Tanzania

## Abstract

**Background:**

Reproductive, maternal, newborn, child health, and nutrition (RMNCH&N) data is an indispensable tool for program and policy decisions in low- and middle-income countries. However, being equipped with evidence doesn’t necessarily translate to program and policy changes. This study aimed to characterize data visualization interpretation capacity and preferences among RMNCH&N Tanzanian program implementers and policymakers (“decision-makers”) to design more effective approaches towards promoting evidence-based RMNCH&N decisions in Tanzania.

**Methods:**

We conducted 25 semi-structured interviews in Kiswahili with junior, mid-level, and senior RMNCH&N decision-makers working in Tanzanian government institutions. We used snowball sampling to recruit participants with different rank and roles in RMNCH&N decision-making. Using semi-structured interviews, we probed participants on their statistical skills and data use, and asked participants to identify key messages and rank prepared RMNCH&N visualizations. We used a grounded theory approach to organize themes and identify findings.

**Results:**

The findings suggest that data literacy and statistical skills among RMNCH&N decision-makers in Tanzania varies. Most participants demonstrated awareness of many critical factors that should influence a visualization choice—audience, key message, simplicity—but assessments of data interpretation and preferences suggest that there may be weak knowledge of basic statistics. A majority of decision-makers have not had any statistical training since attending university. There appeared to be some discomfort with interpreting and using visualizations that are not bar charts, pie charts, and maps.

**Conclusions:**

Decision-makers must be able to understand and interpret RMNCH&N data they receive to be empowered to act. Addressing inadequate data literacy and presentation skills among decision-makers is vital to bridging gaps between evidence and policymaking. It would be beneficial to host basic data literacy and visualization training for RMNCH&N decision-makers at all levels in Tanzania, and to expand skills on developing key messages from visualizations.

**Electronic supplementary material:**

The online version of this article (10.1186/s41256-019-0095-1) contains supplementary material, which is available to authorized users.

## Background

Over the past few decades, the global health community has advocated for increasing the availability, quality, and use of data to inform program and policy decision-making in low- and middle-income countries (LMICs). Coined by some as a “data revolution,” this demand for data is driven, in part, by a need to monitor progress against reproductive, maternal, newborn, and child health and nutrition (RMNCH&N) targets in international accountability frameworks and country-level strategies [1].

Translating data to decision-making is a recognized challenge in global health [2–4]. While capacity for using data is acknowledged as influential, little is known on the statistical capacity and data literacy backgrounds of health decision-makers in LMICs. There are no systematic assessments of data literacy among RMNCH&N decision-makers in LMICs. In the Sustainable Development Goals Report 2017, the United Nations Statistic Division requested improved statistical capacity and data literacy at all levels of decision-making [5]. The World Bank’s Statistical Capacity Indicator, a country-specific composite score that reflects the types and frequency of data collection, does not consider decision-maker data literacy or data use [6].

The role of statistical capacity in decision-making has been explored in some individual countries. In an assessment of strengthening capacity for using data to support policymaking in four countries (Bangladesh, Gambia, India, and Nigeria), researchers found that weak capacity to interpret and use data was a key gap. In the Gujarat, India case profiled by the assessment, only one in ten decision-makers had any previous training in using health data and more than a third of senior program managers “poorly” or “very poorly” use data for decision-making. Studies also describe the complex role of seniority and power dynamics in leveraging data for decision-making – particularly the need to engage senior figures in capacity building and for strengthening institutional capacity [2–4].

Encouraging data-driven action is particularly relevant in Tanzania and similar countries in sub-Saharan Africa that have a wealth of RMNCH&N. One dimension of encouraging data use is ensuring that an audience comprehends the data presented. Different approaches to data visualization—the process of graphically displaying data to tell a story—influence how individuals understand data [7]. There are numerous guidelines for data visualization that aim to help users visually communicate data in a clear and accurate way [8–12]. These guidelines are rooted in aesthetics, human cognition, and short-term memory strengths, and are generally framed as interdisciplinary. Guidelines tend to give less attention to audience data literacy and interpretation capacity.

There is growing literature about whether specific data visualization elements improve comprehension and memorability of visualizations. Edward Tufte, a data visualization pioneer, discourages the use of embellishments like grid lines and images (“chart junk”) and champions visualizations with a high data-ink ratio. The data-ink principle suggests that an optimal visualization only includes essential elements that reflect data [11]. Studies have explored whether chart junk affect understandability. Recent research suggests that chart junk can enhance memorability and comprehension, which conflicts with some data visualization pioneers whom believe chart junk should be avoided [13–16].

Very limited evidence exists on whether current data visualization guidelines influence interpretation and data use by audiences in global health and LMIC settings. In non-LMIC settings, studies on data visualization in the health sector suggest that different data visualization approaches can influence patient and physician interpretation and communication [17–19]. Over the past few decades, the use of geographic information systems (GIS) maps has expanded in clinical settings and global health [20–23]. While GIS maps serve as a powerful tool to highlight population-based trends, there are limited studies on how LMIC data users interpret these maps. Even the most famous example of a map influencing public health decision-making—John Snow’s Broad Street cholera map—did not specifically prompt officials to remove the Broad street pump connected with the cholera outbreak. Officials removed the pump before John Snow drafted the map [23].

Applications of data visualization in African countries is growing. MEASURE Evaluation conducted a study exploring how the United States President’s Emergency Plan for AIDS Relief implementing partners in Namibia, South Africa, Tanzania, and Zambia use different data visualization approaches for HIV programs. The study identified a need to engage end users when developing data visualizations and to train individuals to interpret visualizations [24]. MEASURE Evaluation and Matchboxology also conducted exploratory interviews and workshops with district-level HIV data users in Tanzania and South Africa to identify data visualization solutions for promoting HIV data use at the district-level [25]. Their report also identified insufficient data skills as a barrier to using data for decision-making.

In Tanzania, RMNCH&N decision-making is decentralized and primarily split between the Ministry of Health, Community Development, Gender, Elderly, and Children (MOHCDGEC) and the President Office – Regional Administration and Local Government (PORALG) [26]. As part of the central government, MOHCDGEC provides technical oversight and facilitates development of RMNCH&N strategies. PORALG implements health programs at the zonal, regional, and district levels and coordinates non-governmental organization activities. The Tanzania Food and Nutrition Centre (TFNC) is a government institution responsible for nutrition strategy and policy implementation that works with nutrition-sensitive sectors including both the MOHCDGEC and Ministry of Agriculture. This diverse set of decision-makers positioned to influence RMNCH&N programs and policies can range from politicians with limited health or data expertise to physicians with advanced public health training.

As investment in RMNCH&N data visualization tools and strategies expand in Tanzania and other LMICs, it is important to consider the data literacy and visualization preferences among RMNCH&N decision-makers. This study seeks to address knowledge gaps about RMNCH&N data visualization interpretation capacity and preferences in Tanzania to better inform dissemination of RMNCH&N results.

## Methods

### Research design

We used qualitative methods to characterize the data visualization interpretation capacity and preferences of public-sector RMNCH&N decision-makers in Tanzania. We aimed to use findings to help develop recommendations on improvements to data visualization techniques to promote the use of RMNCH&N data for decision-making in Tanzania. We also conducted this study to support dissemination of findings from the National Evaluation Platform (NEP)‘s evaluation of Tanzania’s One Plan for Maternal Child Deaths in Tanzania (2008–2015) (“One Plan”). Funded by the Government of Canada, the purpose of NEP was to equip government decision-makers with skills to critically evaluate RMNCH&N programs and policies in Malawi, Mali, Mozambique, and Tanzania. The NEP teams in each country that conducted and presented analyses were primarily composed of junior and mid-level civil servants [27].

### Participant recruitment

We recruited 25 participants from MOHCGDEC, PORALG, and TFNC using a snowball sampling strategy and starting with individuals recommended by members of the NEP technical task team (TTT) [28]. Participants eligible for the study had to be employed by MOHCGDEC, PORALG, or TFNC at national level and had to be responsible for approving RMNCH&N program and policy changes (“RMNCH&N decision-makers”). As the NEP TTT includes RMNCH&N technical staff, NEP TTT members provided some initial suggestions for participants. We did not include NEP TTT members as study participants because of their training in statistics and data visualization through the NEP project. We received other suggestions of participants directly from the three institutions and initial respondents. We used a maximum variation sampling strategy to obtain a purposive sample representing different RMNCH&N focus areas, seniorities, and genders. This sample was to reflect NEP’s target audience of a range of civil servants with varying levels of influence and responsibilities in decision-making.

### Data collection and ethics review

In August 2017, we conducted semi-structured in-depth interviews (IDI) with participants in Dar es Salaam and Dodoma. The NEP Tanzania team at the National Bureau of Statistics (NBS), NEP Tanzania Resident Advisor, and a Dar es Salaam-based independent qualitative research consultant conducted all interviews in Kiswahili. A lead interviewer and a note taker conducted each interview, and a student intern from the Eastern Africa Statistical Training Center (EASTC) served as a supplemental note taker during most interviews. Prior to data collection, all members of the data collection team received training in qualitative research methods and data visualization principles.

The interview guide probed participants on their background in statistics, use of RMNCH&N data, understanding of basic statistical concepts, challenges when visualizing and interpreting data, and data visualization best practices for influencing RMNCH&N policy and program changes. We developed the interview guide in English, which we then translated to Kiswahili. The interview guide is available as Additional file 1.

#### Data visualization examples used

During Activity one, we asked participants to come up with key messages for five different visualizations (Table 1). All five visualizations depicted different data from NEP’s preliminary One Plan evaluation results, and featured visualization types that could be made using either Excel or R statistical package. Interviewers provided no help with interpretation.

**Table 1:** Activity 1 data visualization examples and justification for inclusion

Card	Description of key message	Type of visualization	Justification
1	Comparison of proportion (part of a whole)	100% stacked bar chart	People have an easier time interpreting perpendicular angles and segment lengths, so 100% stacked bar graphs are a superior option over pie charts to visualize proportion [7, 8].
2	Trend over time with target	Line graph with target	A line graph is the simplest way to visualize change over time and humans have an easy time judging changes in slope [7, 8, 11, 12]. Target is marked at one point to increase data-ink ratio [11].
3	Comparison of proportion	Stacked bar chart	People have an easier time interpreting perpendicular angles and segment lengths, so 100% stacked bar graphs are a superior option over pie charts to visualize comparisons of proportions [7, 8]. A stacked bar is one technique used to visualize results modelled by the *Lives Saved Tool (LiST)* [35, 36].
4	Trend over time with uncertainty	Line graph with confidence interval bars	A line graph is the simplest way to visualize change over time and humans have an easy time judging changes in slope [7, 8, 11, 12]. Error bands is one approach to visualize uncertainty [8, 34].
5	Geographic performance	Maps	Maps are used to represent geospatial data [8, 11, 12].

In Activities two and three, we explored data visualization preferences by asking participants to sort cards with different visualizations (Table 2). Card sorting is a human-centered design and cultural anthropology technique to identify traits most important to participants on a topic [29]. For each of these activities, we showed participants three different sets of cards, each set included one card with a key message and several cards with different visualizations produced using the same data. We asked participants to rank visualizations in each set based on which most clearly communicated the key message provided. Interviewers did not provide participants with any assistance ranking cards. Table 2 provides rationale for why particular visualizations were selected for inclusion in the ranking activity.

**Table 2:** Activities 2 & 3 data visualization examples and justification for inclusion

Set	Card	Description of key message	Type of visualization	Justification
Activity 2				
1	1	Comparison by wealth quintile (equity groups)	Bar chart	Common approach towards visualizing categories of data.
	2		Dot plot	Coined as “equiplots” by the International Center for Equity in Health at the University of Pelotas, dot plots are used increasingly in global health to visualize equity [37–40].
	3		Dot plot	Intentionally included because it is very difficult to interpret in the context of the key message.
2	1	Comparison among six groups at two time points	Bar chart	Common approach towards visualizing categories of data.
	2		Slope graph	People have an easier time judging changes in slope and slope graphs are alternatives to bar graphs [7, 8, 11, 12].
	3		Dumbbell plot	People have an easier time interpreting dots on a common plane, and dot plots are alternatives to bar graphs [7, 8]. Dot plots are used increasingly to visualize equity [37–40].
3	1	Comparison of proportions	100% stacked bar charts	People have an easier time interpreting perpendicular angles and comparisons within the same plane. This is advocated as a preferred approach over a comparison of two pie chart [7, 8].
	2		Pie charts	This is the most common approach to comparing proportions. Data visualization research suggest this is more difficult to interpret because people have a harder time accurately comparing angles and wedges [7, 8].
	3		Bar charts	Intentionally included because it is very difficult to interpret in the context of the key message.
Activity 3				
1	1	Trend over time with uncertainty	Line graph with shaded confidence intervals	Alternative approach towards visualizing confidence intervals [8, 34]. Has been use by UNICEF and Countdown to 2030 [41, 42].
	2		Line graph with error bars	Standard approach towards visualizing confidence intervals [8, 34].
2	1	Comparison among groups with uncertainty	Bar chart with error bars	Standard approaches towards visualizing categories of data and confidence intervals [8, 34].
	2		Dot plot with error bars	Dots improve data-ink ratio and are combined with a standard approach towards visualizing confidence intervals [8, 11, 34].
	3		Dot plot with shaded error bars	Dots improve data-ink ratio and are combined with a shaded confidence interval bar given people incorrectly interpret error bars [8, 11, 34].
3	1	Comparison of proportion with absolute numbers	Stacked bar chart	Correctly captures both absolute numbers and proportions.
	2		100% stacked bar chart	Intentionally included because it does not fully capture the key message.

The study received ethical clearance from the Tanzania National Institute for Medical Research and Johns Hopkins Bloomberg School of Public Health. All participants gave written and oral consent in Kiswahili.

### Data analysis

An independent qualitative research consultant transcribed and translated all IDI recordings. We chose a sample of transcripts to cross-validate; other members of the team compared audio-recordings to translated transcripts.

To analyze and synthesize results we applied a grounded theory systematic design supplemented with codes developed a priori from the interview guide. Developed by Barney Glaser and Anselm Strauss in 1967, grounded theory is focused on developing a theory to explain a practice or build a conceptual framework that is rooted from data [30]. The grounded theory coding process traditionally involves three stages of coding: open, axial, and selective coding [31]. In our study, the lead investigator first open coded several transcripts. This process involved assigning codes to reoccurring concepts and examples. During the axial coding phase, the lead investigator further broke down categories into sub-categories and grouped related codes. The lead investigator finalized the codebook after integrating additional codes based on the interview guides and card sorting exercises. The lead investigator and a study member coded all transcripts using this codebook and Dedoose qualitative analysis software version 7.6 [32]. After coding all transcripts, the lead investigator used the framework analysis method to organize coded excerpts into broader themes and explore data by participant characteristics [33]. We included all transcripts in the analysis.

We primarily collated visualization rankings from notes taken by the notetakers. When there were discrepancies among rankings in notes, the lead investigator resolved discrepancies by reviewing rankings recorded in transcripts and audio recordings. We report the frequencies with which participants ranked each visualization within card sets.

## Results

We interviewed 25 decision-makers involved with decisions related to national health strategy, vaccines, nutrition, and reproductive and child health (RCH) programs. Most participants were either senior or mid-level professionals; we defined professional experience based on position title (“Senior” = Principles and Directors, “Mid-level” = Senior Officers and Program Officers, “Junior” = Officers) (Table 3).

**Table 3:** Participant characteristics

Characteristic	Total (*n* = 25)
Gender	
Male	16 (64%)
Female	9 (36%)
Focus Area	
General health policy/cross-cutting	5 (20%)
Nutrition	8 (32%)
Reproductive, child health, and newborn	8 (32%)
Vaccines	4 (16%)
Professional Experience	
Senior	10 (40%)
Mid-level	11 (44%)
Junior	4 (16%)

### RMNCH&N data use and interpretation

All participants described how data is critical to their day-to-day responsibilities and used for monitoring & evaluation (M&E) of programs and policy performance, advocacy, commodity forecasts, and/or resource allocation. Even though all participants reported relying on data for their work, many participants have not had any training in statistics or data use since graduating from universities. As a mid-level MOHCGDEC participant described:

“Some say…‘send us to training’…how can I send a person to training while I was never trained on data?” (Mid-level, RCH)

Those who received training reported attending workshops about M&E and using the District Health Information System 2 (DHIS 2), Demographic Health Surveys (DHS), and Stata statistical software.

#### Activity 1

**Fig. 1:**
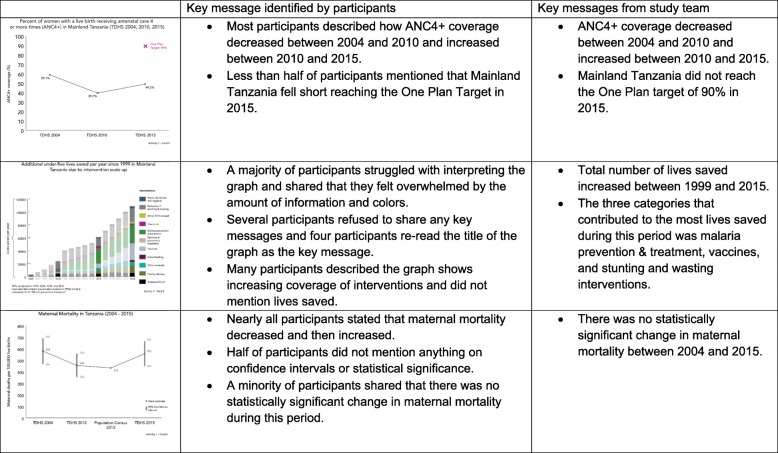
Data visualization interpretation (Activity 1)

Comparison of participants’ key messages to the study team’s key messages suggest that capacity to interpret graphs is mixed (Fig. 1). While most participants correctly described increasing and decreasing trends in both line graphs, many participants did not mention performance against a marked target nor did they explain or discuss the displayed CIs. A marked target in Activity 1 Card 2 represented Tanzania’s One Plan target for women attending 4 or more antenatal visits (ANC4+) of 90%. A majority of participants did not describe the target. Findings differed slightly by respondent characteristics. Among participants who specialize in nutrition, most failed to mention performance against the target. In addition, several mid-level participants did not mention the target. Activity 1 Card 4 focused on changes in maternal mortality since the 2004 TDHS with bars representing 95% CIs. We included this graph given political controversy regarding the 2015 DHS maternal mortality point estimate suggesting that maternal mortality increased since the 2012 population census and 2010 DHS. However, this increase was not statistically significant. Only five participants correctly described that there has been no statistically significant change in maternal mortality between 2004 and 2015. Of participants who did not interpret the CIs, half acknowledged the CIs but did not describe what they meant in the context of the graph. Participants who correctly interpreted the CIs specialized in nutrition, RCH, and vaccines. Nearly all senior level participants did not interpret the CIs.

Participants had the most difficulty interpreting Card 3 – a stacked bar graph depicting results from a *Lives Saved Tool (LiST)* analysis displaying lives saved between 1999 and 2015 due to RMNCH&N interventions.

“It is very congested! What do I have to interpret here? I do not get a message here I just see it [as] confusing.” (Mid-level, RCH)

“There is no key message here. It will bother me to read because separating these small colors. I am color blind. Let’s agree first that there is no key message here. There is many information cluttered in this single chart. It is telling me lives saved, but there is no key message here.” (Mid-level, Nutrition)

Numerous participants reiterated these statements and felt that the graph included too much information and too many colors. Some described the graph as overwhelming and several refused to share any key messages. Other graphs from Activities 2 and 3 are in Additional file 2.

### RMNCH&N data visualization preferences

Participants identified four key factors when deciding how to visualize data.

#### Audience

Participants most frequently cited audience as the main factor when deciding a type of data visualization. Data is prepared for a diverse audience, and participants acknowledged that difference audiences have varying education levels and motivations. In terms of audience motivations, participants articulated that they can foresee the types of questions an audience may ask or data they will want to see. One respondent described how he creates visualizations that show vaccine coverage of different doses because his audience is specifically interested in comparing coverage across doses.

#### Simplicity and understandability

Simplicity and understandability are underlying principles that drive many participants’ data visualization choices, however, there are differing opinions on what types of visualizations are considered “simple” and “understandable.” Participants stated they choose the simplest visualization that can be easily understood, which some described as related to the statistical capacity of the audience. Whether the audience truly understands is unclear to some participants; participants shared that often there is no feedback or only questions on data source asked following a presentation, so they assume the audience understands results received.

“My intention is to make them understand, not give them an exam for them to fail. I use simple methods that I know they will understand at the end of the day.” (Mid-level, RCH)

Nearly all participants described bar charts, pie charts, and maps as visualizations that are easily understood. Icons, words, and line graphs are also used to convey data. When asked about their early experiences learning how to present data, participants shared that they learned to present data in pie charts, bar charts, and tables. Some participants described tables as easy to understand, whereas others felt that tables are only for technical audiences because interpretation is not intuitive.

“To a politician if you use a bar chart, they can easily understand a bar that is long and short. Even with pie charts they can see rounds and segments and get a certain meaning.” (Senior, Vaccines)

“If I am talking to people who are a bit educated, it is good to present through bar and pie charts as they do understand. For those who are less educated like common citizens, using words can be easier for them to understand than pie and bar charts. Telling common citizens and politicians deaths in absolute numbers rather than ratios is easier for them to understand.” (Senior, RCH)

“The most difficult to understand are statistical tables. If you use those statistical data alone, it is challenging for people to read. Because many of them have low understanding on statistical data…many of them are not taught data interpretation so it becomes very difficult. With graphs it becomes simple for them – ‘Ah so this means this.’” (Junior, General health policy/cross-cutting)

Participants also commented that they choose a visualization type that they feel confident and knowledgeable about, so they can facilitate audience understanding.

“I choose a way which is easy for me to interpret the data. I can’t say that I would use a way that I am not experienced [with] or knowledgeable [about] so that I would fail to present the data.” (Mid-level, RCH)

Intentionally limiting the amount of data depicted within a single graph and using strategic formatting are additional techniques used by some participants to promote comprehension. Participants articulated that graphs depicting multiple indicators can be challenging for less technical audiences to interpret.

“Graphs are easy to present when they show data separately instead of combining [indicators]. Showing data combined confuses the audience and presenter.” (Junior, Nutrition)

Participants explained that they use specific fonts and colors (red, yellow, and green) to highlight performance since these colors translate to audiences regardless of statistical background.

#### Information type

Some participants stated that they choose a visualization based on key messages they want to convey. For example, participants mentioned using pie charts to depict proportion, bar charts to show trends over time, and tables and maps to show trends by regions.

Interviewers probed participants on their sense of audience comfort and knowledge of more technical concepts such as proportion and statistical significance. Participants described proportion as a challenging concept to some audiences, and while some audiences are interested in seeing proportions, others are only interested in absolute numbers. There were conflicting views on whether this preference is determined by the audience’s statistical capacity. Respondents acknowledge that an audience’s statistical capacity influences whether depicting CIs is important. Most participants shared that policymakers have a very limited understanding of CIs and described CIs as an “academic” concept. Many participants reported that they rarely see CIs depicted in presentations. A few participants questioned whether depicting CIs has any policy impact.

“I don’t like [confidence intervals] because [it] does not help much…saying you measured confidence intervals will [not] help you to change the policy.” (Senior, Vaccines)

As one participant suggested below, speaking about CIs to a policymaker can be challenging:

“You know our people do not have time…you find a policymaker has [many] meetings so starting to tell them confidence interval stories…I think you will just be pouring water in the sack.” (Mid-level, Nutrition)

Participants rarely provided an accurate definition of CIs. Several commented that being asked about CIs was like being asked to go back to school.

### Graph preferences

Results from Activities 2 and 3 consistently show that participants did not give their highest ranking to the “best” data visualization option as defined by data visualization guidelines. Rather they suggest that familiarity with certain types of visualizations and/or incomplete knowledge of more theoretically effective visualizations may influence preferences. Within each set of cards, participants usually ranked any bar graph or pie chart options highest, regardless of the key message.

#### Activity 2

**Fig. 2:**
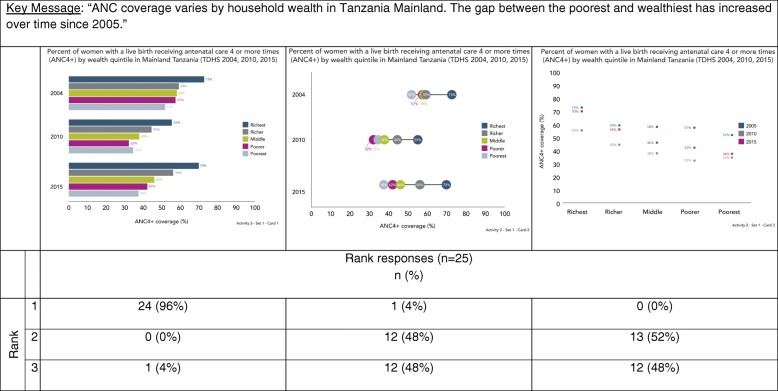
Data visualization ranking by key message – antenatal coverage by wealth quintile (Activity 2)

Figure 2 shows the first card set from Activity 2, which illustrates how the gap in ANC4+ coverage between the poorest and wealthiest households increased between 2005 and 2015. Most participants (96%) ranked the bar chart (graph one) the highest. Participants described the bar chart as the option that is the easiest to understand and could be further improved by orienting the bars vertically. Participants described the dot plot (graph two) as confusing.

**Fig. 3:**
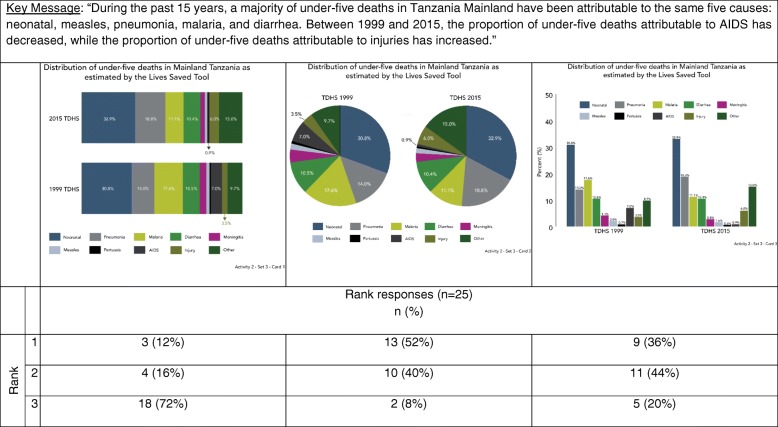
Data visualization ranking by key message – cause of death (Activity 2)

Figure 3 depicts causes of under-five deaths in Mainland Tanzania, as modelled by *LiST*. The key message highlights the top causes of under-five deaths as well as changes over time in the proportion of under-five deaths by cause. Participants ranked the pie chart (graph two) the highest and the 100% stacked bar chart (graph one) the lowest. This set is the only set that contained exclusively bar and pie chart options – both graph types that participants overwhelmingly prefer. Participants felt that the 100% stacked bar chart was difficult to understand, despite this being a type of bar chart:

“Maybe it’s my orientation because I am used to bar and pie charts. Quickly I can’t see a thing.”(Mid-level, RCH)

#### Activity 3

**Fig. 4:**
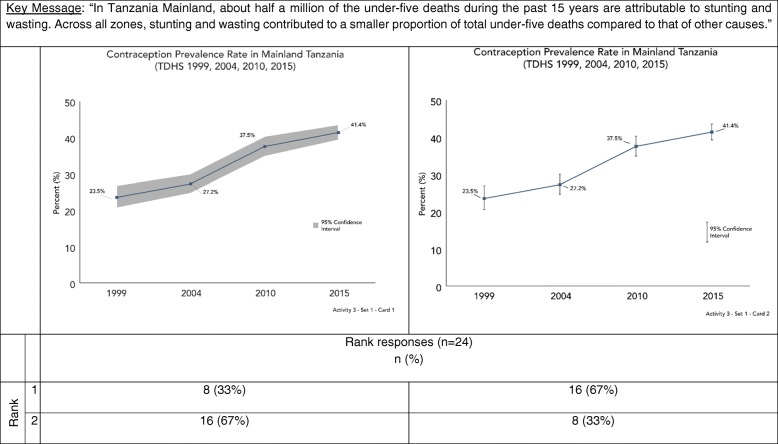
Data visualization ranking by key message – confidence intervals (Activity 3)

Activity 3 featured two card sets that explored approaches to visualizing CIs. Participants generally preferred error bars over shaded regions to represent CIs. Figure 4 shows one card set from Activity 3, which depicts a statistically significant increase in contraceptive prevalence between 2004 and 2015. Most participants chose the graph depicting CIs with error bars (graph two) over the graph depicting CIs with shading (graph one). However, several participants commented that there was no difference between the two graphs, but chose a graph because the exercise required them to rank the graphs.

**Fig. 5:**
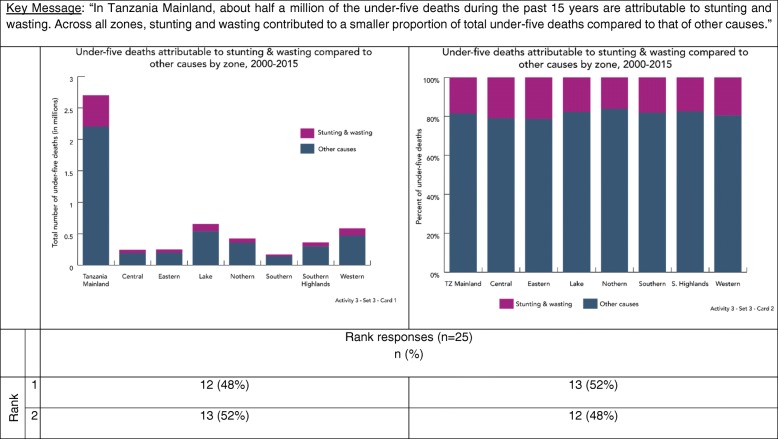
Data visualization ranking by key message –proportion (Activity 3)

Figure 5 shows a card set illustrating two different methods of presenting a proportion. The key message includes both an absolute number and proportion of under-five deaths due to stunting and wasting. Participants ranked the 100% stacked bar graph (graph two) higher, however, the regular bar graph (graph one) is the only option that shows both the number of deaths and proportion.

### Challenges

Participants described several key challenges to visualizing and communicating RMNCH&N data in Tanzania. The greatest challenge flagged by participants is the limited statistical capacity of policymakers. Participants mentioned that the policymakers they present to struggle with interpreting data and are reluctant to hear “statistical jargon.”

“The greatest existing challenge that I see is that some policymakers do not have the knowledge to interpret or present data so it creates controversy in decision making. You can meet a decision maker who gives a statement that jeopardizes people, and it has some influence because of the popularity of that person. However, that person did not give consideration to the data and its meaning, so a decision-maker’s understanding is sometimes an issue. This means we have to do extra work in data presentation – how do we make our policymakers and decision-makers translate data before making decisions.” (Senior, Nutrition)

As presenters, participants shared that they need to have a certain level of statistical knowledge and skills to design an appropriate visualization. They did not, however, identify whether they had this knowledge. Participants felt that presenters should be capable of accurately explaining data to an audience and answering any related questions. Understanding how to present and provoke different audiences is a reoccurring challenge.

“The challenge is that you must understand data analysis, so you can present to an audience with different levels of understanding. You can start presenting your percentages and everyone is sitting there with no questions. Higher-level people cannot tell you ‘I do not understand you.’” (Mid-level, RCH)

Another challenge is distrust in data presented. Participants mentioned how audiences have questioned the validity of data presented, particularly if there is a lack of understanding how data were collected and the data source. Policymakers have been reluctant to accept data if the data suggest unfavorable results. For example, when the DHS 2015/16 reported an increase in maternal mortality, policymakers did not want to accept the fact that maternal mortality increased. This led to discussion on what should be considered the “true” maternal mortality ratio.

“Another problem that I see is that people don’t believe in statistical data. You can present data, but you find a leader or politician saying this data is not right! The success of politicians [can be] based on data quality.” (Senior, RCH)

Finally, participants discussed how there are many other factors beyond visualizing and communicating data that influence whether RMNCH&N data can be translated into policy. Even the most compelling data may not inspire change if there is insufficient funding and human resources to facilitate policy and programmatic changes, and weak political will. To help mitigate these barriers, participants reinforced that data presented must be tied to specific policy timelines.

### Suggestions for best practices

Participants’ suggestions on best practices when visualizing and communicating RMNCH&N data fell into two domains: (1) formatting and presentation and (2) training for data visualization producers and consumers (Table 4). Concise products are preferred to lengthy reports. Participants recommended using simple language in products and writing key messages directly on graphs. Nearly all participants also mentioned color preferences including using red, green, and yellow to illustrate trends, choosing color palettes that are color blind friendly and have distinct number of bold colors, and limiting the total number of colors. Participants also shared other specific aesthetic preferences like including grid line backgrounds and a legend on graphs. Many participants requested training on basic data literacy and data visualization. Participants expressed eagerness to improve their capacity to present data to policymakers.

**Table 4:** Suggestions for improving data visualization for RMNCH&N

Domain		Suggestion
Formatting and presentation	Graph formatting	• Use vertical bar graphs rather than horizontal bar graphs • Label values directly on graphs • Include a key with all graphs • Include grid line backgrounds on graphs • Avoid including several indicators within a single graph
	Color	• Use colors that easily represent issues (red, green, yellow) • Limit the total number of colors and choose distinct, bold colors • Use color blind-friendly colors
	Structure	• Include short interpretations (key messages) adjacent to graphs • Use “simplest” graph possible to visualize data and ensure associated messages are understandable and written in simple language • Create succinct materials rather than long reports
Training		• Develop curriculum on basic data literacy and statistics, data visualization, and data presentation skills for policymakers

## Discussion

To our knowledge, this is the first known study on data visualization interpretation and preferences among government RMNCH&N decision-makers in any LMIC. The results from our study suggest that data interpretation skills are mixed among RMNCH&N decision-makers in Tanzania and visualization preferences do not align with current data visualization guidelines. Although data is a critical aspect to all participants’ jobs, capacity to comprehend visualizations and identify key messages varied among participants at all professional levels and focus areas.

Edward Tufte, a pioneer of data visualization, warns against underestimating the intelligence of the audience receiving a data visualization [11]. However, there is an additional risk with overestimating the capacity of an audience. Although participants in this study shared that there is limited audience feedback following presentations of RMNCH&N data, this does not mean they understand what is presented. Rather, this silence could reflect limited comprehension and/or a reluctance to draw attention to themselves. Most surprisingly are the numerous participants who brought up color blindness, which is not usually raised when RMNCH&N data is presented or routinely considered when producing RMNCH&N data products in LMICs.

This study does not explore specific roles of individual civil servants in the decision-making process. In Tanzania, this role can vary based on institution and expertise. However, given that individual responsibilities can vary across the government, basic data literacy and visualization training for RMNCH&N decision-makers at all levels would be valuable to promote comprehension of RMNCH&N data visualizations. This would especially be beneficial for national and subnational policymakers and politicians who often have a very limited background in statistics, but still play a major role in setting RMNCH&N priorities across Tanzania. This recommendation is aligned with findings from other studies on building institutional capacity to promote data for decision-making [2–4]. NEP’s intention was to disseminate findings in a way that would pass a “front page test” – a format where anyone would be able to interpret findings on their own. In response to this study’s findings, NEP Tanzania conducted four workshops with NBS, MOHCGDEC (RCH), PORALG, and TFNC staff on basic data literacy and visualization in June 2018.

Participants’ visualization rankings largely did not align with data visualization research and field best practices. For example, data visualization principles promote the use of dot plots to visualize equity since space between dots on a common axis can easily be interpreted, however, respondents ranked dot plots lower than other options [7, 8]. Given that dot plots are increasingly used by global health stakeholders to visualize equity, it is important to ensure that the audience can accurately interpret these types of graphs. Similarly, while participants ranked CIs depicted as error bars higher than CIs depicted as shadows (Fig. 4), however, research suggests that using error bars depicting CIs can be interpreted inaccurately [34]. When depicting CIs is necessary to accurately interpret a key message, like the trend in maternal mortality in Tanzania, presentation of CIs becomes increasingly important.

This study illustrates a tension between visualization approaches that are “familiar” to target audiences compared to more novel approaches championed by the data visualization field. Even though participants demonstrated a clear preference for bar graphs and pie charts in the study, this should not be interpreted as a recommendation to only use these types of visualizations for RMNCH&N data. Participants still struggled to correctly and comprehensively identify key messages of these more familiar visualization types. Participants expressed preference for vertical over horizontal bar charts, grid lines, legends, and other visualization embellishments that some data visualization experts dismiss as chart junk or reducing the data-ink ratio. These preferences may also reflect a preference for familiar graph elements or limited data visualization training. The aspect of familiarity is rarely addressed in the data visualization field, and it is worth exploring how to balance data visualization best practices with personal preferences rooted in familiarity if the end goal is data comprehension.

Participants’ discomfort interpreting 100% stacked bar charts, dot plots, slope graphs, and other types of visualizations included in this study suggest that we cannot assume RMNCH&N decision-makers can understand these visualization types. When these types of visualizations are used, presenters should make a concerted effort to guide audiences through interpreting these graphs. Similarly, participants’ difficulty understanding CIs suggests that if included in a visualization, presenters must provide an adequate interpretation for audiences. The results additionally highlight that it may be unwise to assume any RMNCH&N audience has an innate ability to interpret unfamiliar graphs like equiplots – an important consideration as use of these visualizations expand in global health.

Finally, as this is the first known study of data visualization interpretation capacity and preferences in global health and LMICs, it would be valuable to conduct similar studies in other regions to explore consistencies in findings. This is particularly important given large investments in data for global health decision-making in LMICs.

### Limitations

This study only includes a subset of all government RMNCH&N decision-makers in Tanzania and results may not be generalizable. Snowball sampling of participants may also be inherently biased and not a representative sample. Since we conducted interviews in Kiswahili, it’s possible that some participants’ responses and visualization interpretations could have been omitted when translated to English. Furthermore, since the sample size is small, any findings by participant characteristics are not conclusive.

RMNCH&N programs and policies in Tanzania are influenced by many stakeholder groups not included in this study. We intentionally focused on MOHCDGEC, PORALG, and TFNC, however, academic and more statistics-focused government institutions can influence RMNCH&N policies. Data literacy and visualization skills may be higher among individuals that are in academia or in government institutions like NBS. Since RMNCH&N program implementation and policymaking overlap in Tanzania, and we cannot disaggregate results by these ambiguous roles.

Finally, data used for activities in this study do not represent all possible RMNCH&N data or visualization types. Given that the intention of this study was to influence how we presented results from NEP’s One Plan evaluation and NEP’s focus on capacity building, we used a selection of relevant RMNCH&N data and visualization types that could theoretically be made using software available to the team.

## Conclusions

Creating an environment of evidence-informed RMNCH&N policy and programs requires more than inundating decision-makers with data. This study concluded that data literacy and visualization skills among RMNCH&N decision-makers in Tanzania are variable. Decision-makers must be able to understand, interpret, and communicate RMNCH&N data. While the field of data visualization suggests principles that theoretically improve communication of data, another aspect that must be considered is the audience’s own familiarity and comfort with certain types of visualizations and formatting. Although participants in this study overwhelmingly prefer bar graphs and pie charts, there is an opportunity to build capacity in using other types of graphs, especially as other types of visualization approaches are adopted by global accountability frameworks and health initiatives.

## Additional files

Additional file 1: Interview Guide. (DOCX 23 kb)

Additional file 2: Additional visualizations used in study. (DOCX 295 kb)

## Abbreviations

ANC4 +: Attending 4 or more antenatal visits
CI: Confidence interval
DHIS 2: District Health Information System 2
DHS: Demographic Health Surveys
EASTC: Eastern Africa Statistical Training Center
GIS: Geographic information systems
IDI: in-depth interviews
LiST: Lives Saved Tool
LMICs: Low- and middle-income countries
M&E: Monitoring & evaluation
MOHCDGEC: Ministry of Health, Community Development, Gender, Elderly, and Children
NBS: National Bureau of Statistics
PORALG: President Office – Regional Administration and Local Government
RCH: reproductive and child health
RMNCH&N: Reproductive, maternal, newborn, child health, and nutrition
TFNC: Tanzania and Food and Nutrition Centre
TTT: Technical task team
