# Supplementary material for: Optimizing data visualization for reproductive, maternal, newborn, child health, and nutrition (RMNCH&N) policymaking: data visualization preferences and interpretation capacity among decision-makers in Tanzania
Source: Glob Health Res Policy. 2019 Feb 15;4:4. doi: 10.1186/s41256-019-0095-1 (PMC6376719; doi:10.1186/s41256-019-0095-1)
Supplement: Supplementary file 1 — Interview Guide. (DOCX 23 kb) [file 41256_2019_95_MOESM1_ESM.docx]

# Additional file 1: Interview Guide

NATIONAL BUREAU OF STATISTICS | NATIONAL EVALUATION PLATFORM

MRADI WA NATIONAL EVELUATION PLATFORM I OFISI YA TAIFA YA TAKWIMU

PARTICIPANT QUESTIONS

MASWALI YA WASHIRIKI

**Study Title:** Visualizing and communicating maternal, newborn, child health, and nutrition (MNCH&N) data

**Jina la Utafiti:** Uwasilishaji wa taarifa za kitakwimu za afya ya kinamama, watoto wachanga na wadogo pamoja na lishe

**Principal Investigator/ Mkaguzi Mkuu**: Tricia Aung

**IRB No./ Namba**: JHU/IRB00007617; NIMR/HQ/R.8a/Vol. IX/2434

**Version Date/ Tarehe ya toleo**: 27 June 2017

INTRODUCTION

UTANGULIZI

Welcome and thank you for participating in this interview. The purpose of today’s discussion is to explore your thoughts on data visualizations for Maternal, Newborn, Child Health, and Nutrition (MNCH&N) policymakers. I will ask you general questions about your viewpoints, and I am interested in hearing about the topics mentioned. The IDI will last no more than an hour. Do you have any questions?

**Swali:** Karibu na asante kwa ushiriki wako katika majadiliano haya. Lengo la mazungumzo yetu ni kupata mawazo yako katika maswala ya uwelewa wa taarifa za afya ya kinamama, watoto wachanga na wadogo pamoja na lishe kwa watunga sera. Nitakuuliza maswali kuhusu maoni yako na nitafurahi kusikia majibu yako katika maswali nitakayo uliza. Mazungumzo yetu hatamaliza saa moja. Je una swali lolote ?

SECTION 1

SEHEMU 1

**Question 1:** I am now going to ask a few questions to get a better sense of your work. What is your current role? How many years of you been in your current role? How do you currently use data in this role? Have you had any formal or informal (e.g. on-the-job) training in data use or statistics? If so, please describe.

**Swali 1:** Sasa ninaenda kukuuliza maswali machache ili kujua vyema kazi yako. Kwa sasa unafanya kazi gani?Je una muda gani katika nafasi yako ya sasa hapa kazini? Je unatumiaje takwimu katika nafasi yako kwa sasa?Je umewahi kuchukua mafunzo ya takwimu chuoni au kazini? Kama ndio tafadhali eleza.

**Question 2:** Think about when you first started learning about data and statistics. For the purposes of this interview today, when we refer to “data,” we are describing quantitative information that can be used for analysis. What were different ways that you were taught to visually present data? (For example in a report or when you made a presentation with slides)

**Swali 2:** Fikiria mara ya kwanza ulipoanza kujifunza kuhusu data na takwimu. Kwa ajli ya mahaojiano haya ya leo tunaposema data tunaelezea upimaji wa taarifa zinazoweza kutumika katika uchambuzi. Je ulijifunza njia -zipi za kuwasilisha taarifa zako za kitakwimu katika vielelezo mbalimbali? (kwa mfano kwenye ripoti au unapotengeza presentation kwa slides)

**Question 3:** *(after interviewee lists examples, follow up with probing questions)* When you learned about displaying data by different ways like [list examples that the interviewee mentioned in response to question 1] how do you decide when to use certain types of visuals?

**Swali 3:** (Baada ya mshiriki kutaja njia mbalimbali zinakili na endelea kumdodosa) Wakati ulipojifunza kuwasilisha taarifa katika njia tofauti tofauti kama (taja njia mojawapo aliyotaja katika mfano kwenye swali la kwanza) kitu gani kinakusaidia kuamua ni aina gani ya kielelezo utumie wakati fulani?

**Question 4:** In your current job, what kind of materials do you prepare or review that have these types of data visuals that you mention?

**Swali 4:** Katika kazi yako ya sasa ni taarifa zipi unatengeneza au unazirejea ambazo zina baadhi ya maumbo / vielelezo ambavyo umetaja?

**Question 5:** When you present MNCH&N data, who are the main audiences? For example, your supervisors (identify what their position is), MOH policymakers, health facility staff, development partners, NGOs, etc.

**Swali 5:** Je wakati unawasilisha taarifa za tarifa za afya ya kinamama, watoto wachanga na wadogo pamoja na lishe ni nani wasikilizaji wako?Mfano wakuu wako wa kazi (tutajie cheo chao) watunga sera wa Wizara ya afya, watumishi katika vituo vya afya, wadau wa maendeleo, mashirika yasiyo ya kiserikali n.k?

ACTIVITY 1

**Question 6 (Activity 1):** I am now going to show you a printed set of tables and graphs that visualize different trends in MNCH&N in Tanzania. The data for these visuals represent national and region-level data from TDHS household surveys and TSPA. Based on what you see, please describe what the key messages are for each visual. [Show the cards one at a time].

**Swali 6:** Sasa naenda kukuonyesha majedwali na maumbo yanayoonyesha mwelekeo wa taarifa za afya ya kinamama, watoto wachanga na wadogo pamoja na lishe Tanzania. Taarifa hizi zinawasilisha takwimu za kitaifa na mikoa kutoka katika tafiti za kaya za TDHS and TSPA. Kutokana na utakachoona hapa naomba uniambie ni ujumbe gani mahususi uliopo katika vielelezo hivi (muonyeshe kadi)

SECTION 2

SEHEMU 2

**Question 7:** Within your current organization, who is deciding how data is represented for dissemination?

**Swali 7:** Katika sehemu yako ya kazi kwa sasa ni nani ana mamlaka ya kusema takwimu ziwe kwenye maumbo gani kabla ya kuwasilisha kwa walengwa?

**Question 8:** What factors do you consider when deciding what type of visual you will use to display data? Do you consider multiple ways to represent the data?

**Swali 8:** Mnatumia vigezo gani kuamua ni aina gani ya maumbo yatumike kuonyesha takwimu zenu? Je huwa mnatumia mbinu mbalimbali katika kuwasilisha takwimu zenu?

**Question 9** When was the last time you presented data (either a presentation or a report) to MNCH&N policymakers? What was the purpose of your presentation? What type of background experience and training do you think your audience had in statistics and working with MNCH&N data?

**Swali 9:** Lini ilikuwa mara yako ya mwisho kuwasilisha data (taarifa au repoti) kwa watunga sera wa maswala ya mama na mtoto? je ni uzoefu upi unadhani wasikilizaji wako walikuwa nao katika takwimu na kufanya kazi na taarifa za kinamama na watoto?

**Question 10:** In response to your presentation, what types of questions or comments did you receive about the data presented? Did you receive any reaction to the types of visuals you presented or requests for clarification?

**Swali 10:** Kutokana na uwasilishaji wako je ni maswali gani na maoni ulipokea kutokana na data ulizowasilisha? Je ulipata oni lolote kuhusu vielelezo ulivyotumia au ombi la kuelezea Zaidi?

**Question 11:** In general, when you present data to MNCH&N policymakers, what types of data visuals do you feel like the audience seems to grasp easily? Are there data visuals that you feel like the audience has a more difficult time understanding? Please describe.

**Swali 11:** Kwa ujumla Unapowasilisha taarifa za kinamama, watoto wadogo na wachanga kwa watunga sera je ni aina gani ya maumbo/vielelezo au uwasilishaji unayodhani washiriki wako wanailewa mapema? Je kuna aina nyingine ya uwasilishaji na vielelezo unavyodhani washiriki wako wanapata shida kuielewa mapema? Tutajie.

**ACTIVITY 2**

**Question 12:** I’m now going to show you 3 different sets of cards that each have 3 different visualizations based on the same data set. I will also show you a key message prepared for each set. Within each set of cards, rank from best to worse the visuals that best represent the key message(s). For the visual you rank the best, please describe how the visual could be further improved. [Show the card sets one at a time].

**Swali 12:** Sasa nitakwenda kukuonyesha seti tatu za kadi zenye vielelezo vitatu tofauti vinavyotokana na data zinazofanana. Pia nitakuonyesha ujumbe mahususi uliotengenezwa kwa ajili ya kila seti. Katika kila seti ya kadi, naomba unipangie ujumbe mahsusi mzuri sana hadi mbaya sana kulingana na kielelezo kilichowekwa. Kwa vielelezo utakavyochagua kuwa ni vizuri sana tafadhali tuambie jinsi gani vielelezo hivyo vinaweza kuboreshwa.

SECTION 3

SEHEMU 3

**Question 13:** What do you think confidence intervals represent? Do you think MNCH&N policymakers understand this concept? Have you seen confidence intervals depicted in MNCH&N data visualizations in Tanzania? Do you think depicting confidence intervals is important to MNCH&N policymakers?

**Swali 13:** Je unadhani confidence interval inawakilisha nini? Unadhani watunga sera wa maswala ya mama, watoto wadogo na wachanga na lishe wanaelewa dhani hii? Je umeshawahi kuona confidence interval inatumika katika vielelezo vya taarifa za kitwakimu za kinamama na watoto Tanzania. Unadhani kuonyesha confidence intervals ni muhimu kwa watunga sera wa maswala ya mama, watoto na lishe Tanzania?

**Question 14:** What is a proportion?

**Swali 14**:

ACTIVITY 3

**Question 14:** I am now going to show you three sets of cards. Each set has 2 or 3 different visualizations based on the same data and a key message. Rank the data visualization cards in the order of which you feel best represents the key message. [Show the card sets one at a time].

**Swali 14:** Sasa naenda kukuonyesha seti tatu za kadi. Kila seti ina aina 2 au 3 tofauti za vielelezo vya takwimu na ujumbe mashsusi unaofanana. Panga vielelezo hivyo katika mpangilio unaodhani unawasilisha vyema ujumbe mahsusi. (Onyesha kadi seti moja baada ya nyingine)

SECTION 4

SEHEMU 4

**Question 15:** What do you think are the great challenges to visualizing and communicating MNCH&N data to policymakers?

**Swali 15:** Unadhani ni changamoto gani kubwa iliyopo katika kuangalia na kuwasilisha taarifa za kitakwimu za kinamama, watoto wachanga na wadogo pamoja na lishe kwa watunga sera?

**Question 16:** What do you think should be included as best practices when visualizing and communicating MNCH&N data to policymakers?

**Swali 16:** Unadhani nini kiongezwe kama kitu bora wakati wa kuangalia na kuwasilisha taarifa za kitakwimu za kinamama, watoto wachanga pamoja na wadogo kwa watunga sera?

**Question 17:** Do you have any other thoughts that you would like to express on data visualization for MNCH&N policymakers?

**Swali 17:** Je una wazo lingine lolote unalotaka kutueleza linalohusiana na kuwasilisha taarifa za kitakwimu zinazohuzu kinamama, watoto wachanga pamoja na wadogo kwa watunga sera?
