# Supplementary material for: Optimizing data visualization for reproductive, maternal, newborn, child health, and nutrition (RMNCH&N) policymaking: data visualization preferences and interpretation capacity among decision-makers in Tanzania
Source: Glob Health Res Policy. 2019 Feb 15;4:4. doi: 10.1186/s41256-019-0095-1 (PMC6376719; doi:10.1186/s41256-019-0095-1)
Supplement: Supplementary file 2 — Additional visualizations used in study. (DOCX 295 kb) [file 41256_2019_95_MOESM2_ESM.docx]

# Additional file 2: Additional visualizations used in study

| **Activity 1** | Key message identified by participants | Key messages from study team |
| --- | --- | --- |
| **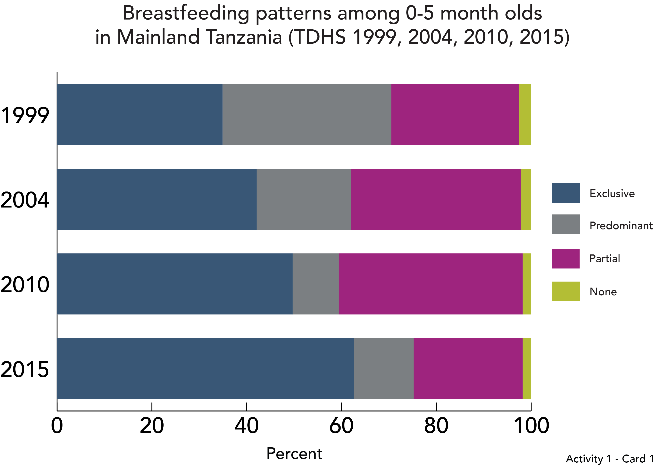** | - Most participants described how exclusive breastfeeding increased between 1999 and 2015. - Several participants also noted changes in the proportion of predominant and partial breastfeeding. - Few participants mentioned how 0-5 month old children not breastfed has remained constant or decreased. | - Proportion of 0-5 month old children that were exclusive breastfeeding increased between 1999 and 2015. - Proportion of 0-5 month old children not breastfed has remained the same. - Proportion of children predominantly breastfed decreased then increased between 2010 and 2015. |
| **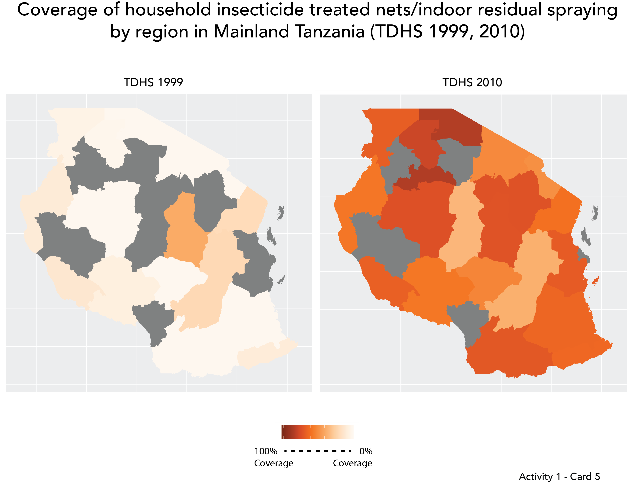** | - Majority of participants described how coverage of insecticide treated nets/indoor residual spraying increased in all regions. - Several participants had difficulty interpreting the colors in the graph – possibly because of color-blindness or not understanding the legend. | - Insecticide treated nets/indoor residual spraying increased dramatically for most regions between 1999 and 2010. |

| Activity 2  Key Message: “About half of women receiving ANC in Tanzania Mainland reported being satisfied with how well a provider met a set of expectations about the services. Client satisfaction does not vary by type of facilities, but is higher in non-government facilities compared to that in government facilities.” | | | | |
| --- | --- | --- | --- | --- |
|  | | 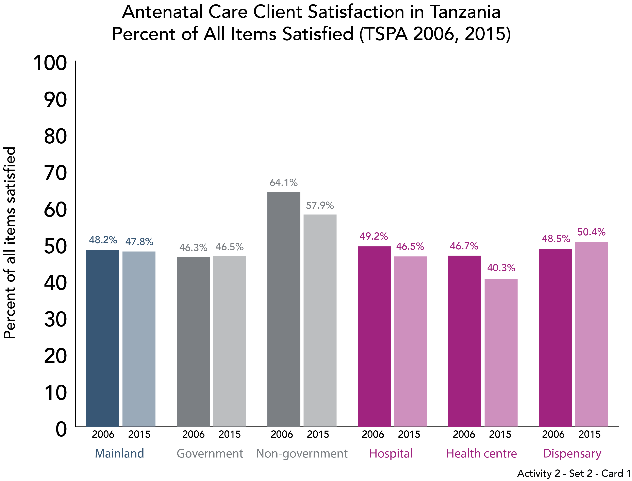 | **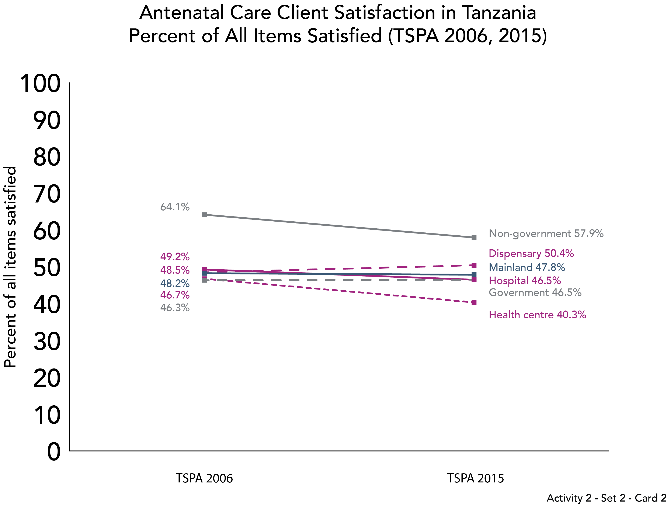** | **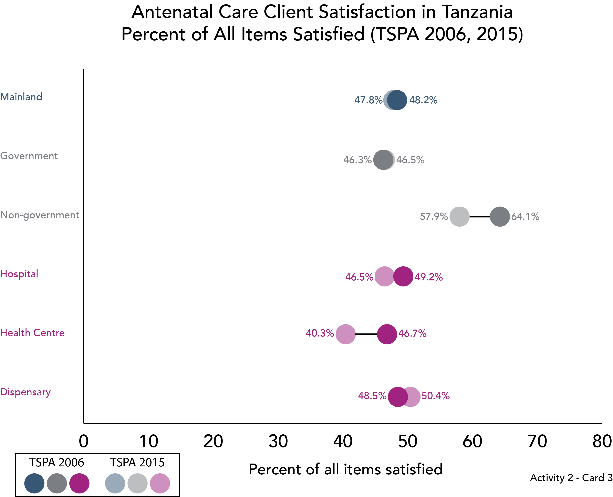** |
|  |  | Rank responses n (%) | | |
|  |  | n=25 | n=24 | n=24 |
| Rank | 1 | **23 (92%)** | 2 (8%) | 0 (0%) |
|  | 2 | 2 (9%) | **13 (54%)** | 9 (38%) |
|  | 3 | 0 (0%) | 9 (38%) | **15 (63%)** |

| Activity 3  Key Message: “ANC readiness—a measure of how well a facility can provide services—varies by region, however, the variation is not statistically significant.” | | | | |
| --- | --- | --- | --- | --- |
|  | | 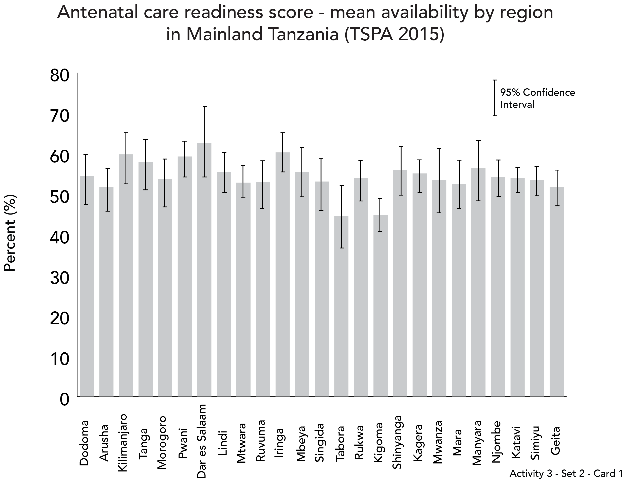 | **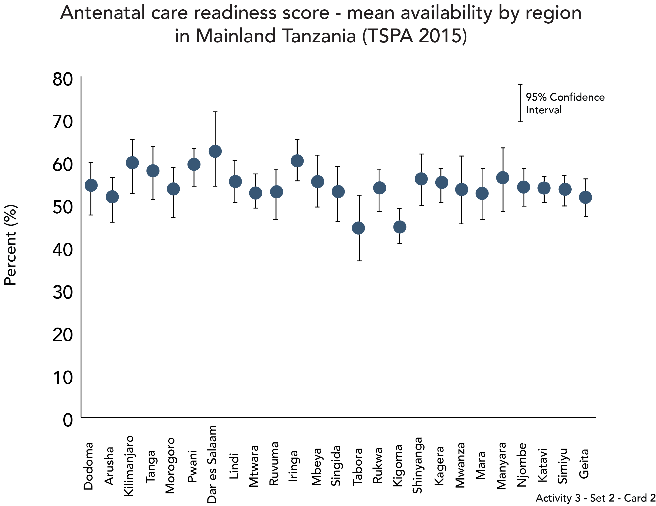** | **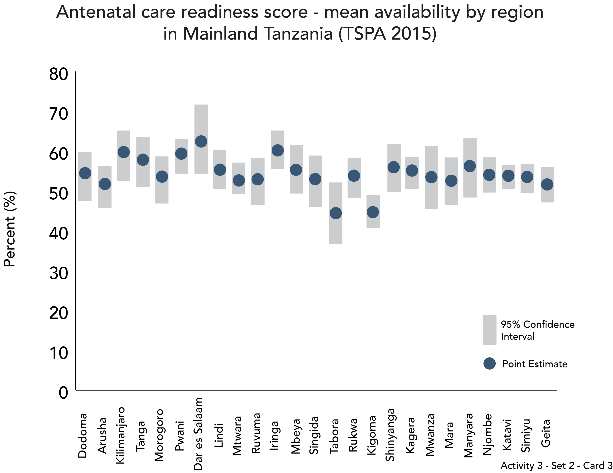** |
|  |  | Rank responses n (%) | | |
|  |  | n=25 | n=24 | n=24 |
| Rank | 1 | **15 (60%)** | 5 (21%) | 5 (21%) |
|  | 2 | 6 (24%) | **12 (50%)** | 6 (25%) |
|  | 3 | 4 (16%) | 7 (29%) | **13 (54%)** |
